# The camelliagenin from defatted seeds of *Camellia oleifera* as antibiotic substitute to treat chicken against infection of *Escherichia coli* and *Staphylococcus aureus*

**DOI:** 10.1186/s12917-015-0529-z

**Published:** 2015-08-18

**Authors:** Yong Ye, Qian Yang, Fei Fang, Yue Li

**Affiliations:** Department of Pharmaceutical Engineering, School of Chemistry and Chemical Engineering, South China University of Technology, Guangzhou, 510640 P R China

**Keywords:** *Camellia oleifera*, Camelliagenin, Antibacterial effect, Bacterial biofilm, Antibiotic substitute

## Abstract

**Background:**

*Escherichia coli* and *Staphylococcus aureus* are the main pathogens infectious to poultry, and their resistances against antibiotics have become troublesome currently. Biofilm formation is an important reason for drug resistance. Our previous research has found that the extract of *Camellia oleifera* seeds has lots of pharmacological effects. In order to find the substitute for antibiotics, the saponin was isolated from the defatted *C. oleifera* seeds with structural identification. Its efficacy was evaluated by the inhibition on amoxicillin-resistant *E. coli* and erythromycin-resistant *S. aureus* and therapeutic effect on chicks infected by the two bacteria.

**Results:**

The bacterial growth inhibition rate increased and the bacterial count *in vivo* decreased significantly in dose dependence after administration of the saponin and its combination with amoxicillin or erythromycin, suggesting its antibacterial effect. The saponin identified as camelliagenin shows significant inhibition on the biofilm of *E. coli* and *S. aureus*, and it is related to the decrease of mannitol dehydrogenase (MDH) activity and extracellular DNA (eDNA) content. Molecular simulation reveals the strong interaction existing between the saponin and MDH or eDNA.

**Conclusions:**

The mechanism of camelliagenin’s improvement on antibiotic effects is its interaction with MDH and eDNA in biofilm. The saponin is a prospective substitute of antibiotics, and molecular simulation is a convenient alternative method to find out hopeful candidates of antibiotics substitute.

## Background

*Escherichia coli* and *Staphylococcus aureus* are major pathogenic bacteria infectious to poultry. *E. coli* causes the colibacillosis such as acute septicaemia, vitelline peritonitis, enteritis, genital diseases, and so forth, its morbidity and lethality are the highest in bacterial diseases of chicks in China [1, 2]. The pathogen is currently treated by antibiotics, but its resistance against antibiotics can be easily acquired, and antibiotics lead to the deficiency of therapeutic effects [3]. *S. aureus* can cause acute septicaemia, arthritis, chick omphalitis, cutaneous necrosis and periostitis, and it is another pathogen leading great economic losses to the animal husbandry in China [4]. It is also easy to acquire drug resistance; especially methicillin resistant *S. aureus* (MRSA) is resistant to most of antibiotics. The resistance against antibiotics becomes great threat to animals and human being, and it is meaningful to find out the antibiotic substitute to reduce the usage of antibiotics. Herbs and plants offer plenty of compounds, which may replace antibiotics with strong antibacterial activities.

*Camellia oleifera,* an evergreen plant, mainly grows in the middle region of China. Its seeds are used for the extraction of edible oil. The yield of the seeds dramatically increases in recent years because of plant edible oil requirement and cultivation, the production of defatted seeds is up to 800,000 tons per year [5]. Although the defatted seeds are rich in active compounds [6, 7], they have been discarded without isolation and further exploitation. The total flavonoids, saponins and polysaccharides were detected in seeds of *Camellia oleifera* with the contents of 1–3, 10–14 and 15–20 % respectively [8–10]. The effective utilization of them will provide large amount of bioactive products. It is valuable to extract them for industrialization.

Our previous research has found that the extract of *Camellia oleifera* seeds have lots of pharmacological effects such as antioxidation, anti-inflammation and analgesia, which are mainly due to the saponin [11]. It is possible to develop the saponin extract as antibiotic substitute for animal husbandry because of its abundant and cheap resource.

In order to evaluate its effect and discuss the mechanism, the saponin was isolated from the defatted seeds of *C. oleifera* with structural identification. Its efficacy was evaluated by amoxicillin-resistant *E. coli* and erythromycin-resistant *S. aureus* induced infection in chicks. Bacterial biofilm formation is an important reason for the resistance against antibiotics, recurrence and difficulty to control by chemicals [12]. The action mechanism was revealed by inhibitory effect of the saponin on biofilm formation through interaction with mannitol dehydrogenase (MDH) activity and extracellular DNA (eDNA).

## Results and discussion

### Purity of the extract and structure of the purified compound

Natural saponins generally exist in forms of glycosides, and different kinds of glycosides of saponin have been found in seeds of *C. oleifera* [13]. Saponin glycosides are soluble in water and aqueous solvents. The extracts are the mixture of different glycosides, which can be achieved by current technique [14]. HCl aqueous solution with ultrasonic extraction applied in our research can hydrolyze saponin glycosides into sapogenin, which are insoluble in water and easily isolated by precipitation to get the purified products.

**Fig. 1:**
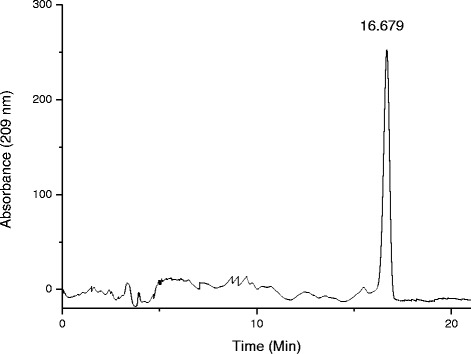
HPLC chromatograph of the saponin extract

Average yield of the saponin extract was (12.5 ± 0.7) % by the separation technique in three repetitions. Purity of the extract was calculated by peak areas in HPLC (Fig. 1). Relative percentage of saponin is (82.3 ± 4.2) %. The yield and purity suggests that the technique is practical and cost effective because the ultrasonic assisted acid-base alternative extraction does not need organic solvent and expensive equipment. Ultrasonic can expedite dissolution in lower temperature, which protects activity of thermosensitive compounds in the extraction [15].

**Fig. 2:**
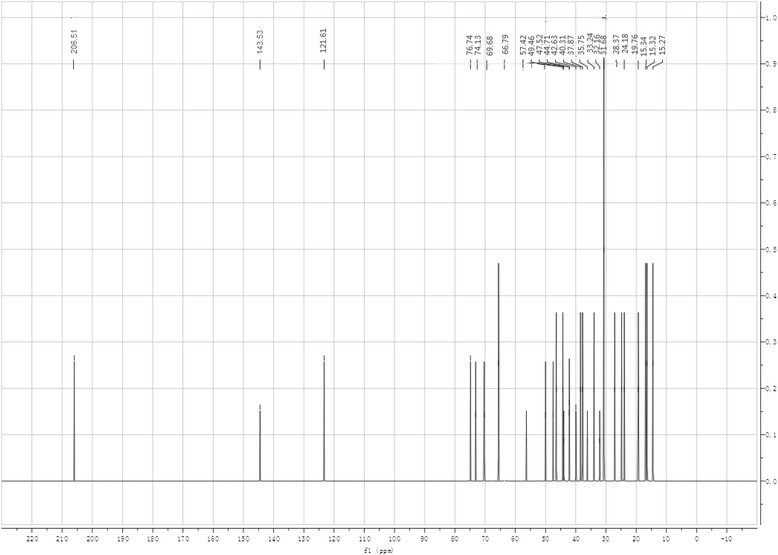
NMR spectra of the camelliagenin

**Fig. 3:**
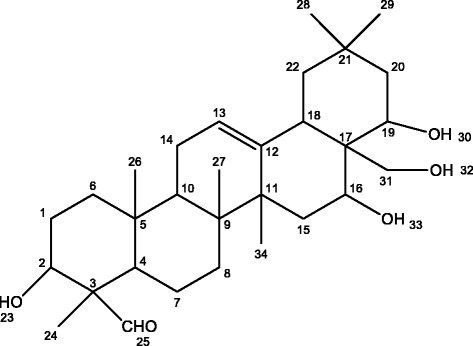
Structures of the saponin isolated from the defatted seeds of *Camellia oleifera*

The purified saponin was amorphous powder, insoluble in water, and soluble in ethanol, acetone and DMSO. mp 251.2–251.7 °C, m/z 488.1 (M+). There was an absorption peak of UV at 207 nm. IR spectra: hydroxyl (3435 cm^−1^), no characteristic absorption of α, β-unsaturated ester and ether. There were one carbonyl (d 9.49, J = 12.48), two olefinic protons (d 8.01, 7.49) but no Tig moiety signal in ^1^H NMR spectra. The signals of ^13^C NMR spectra were shown in Fig. 2 and attributable to the following: δ206.5 (C-25), 143.5 (C-12), 121.6 (C-13), 76.7 (C-19), 74.1 (C-2), 69.7 (C-31), 66.8 (C-16), 57.4 (C-3), 47.5 (C-4, 10, 22), 44.7 (C-17), 42.6 (C-11, 20), 40.3 (C-9, 18), 37.9 (C-5, 6, 15), 31.7 (C-8, 20), 28.4 (C-28, 29), 24.2 (C-14, 34), 15.3 (C-24, 26, 27). It is a sapogenin structure named camelliagenin (C_30_H_48_O_5_), which is consistent with literature [16]. Its structure is shown in Fig. 3.

### Inhibitory effect of the saponin on bacteria *in vitro* and *in vivo*

Inhibitory effect of the saponin on bacteria *in vitro* was measured by MIC_90_ and IC_50_ of the camelliagenin on bacterial growth in culture medium. MIC_90_ of the saponin on 35 strains of *E. coli* and 30 strains of *S. aureus* was separately 71.4 ± 6.3 μg/ml and 94.5 ± 9.7 μg/ml. The camelliagenin had significant inhibition on growth of both *E. coli* and *S. aureus*, but its effect on *E. coli* was stronger than *S. aureus* suggestive of its selectivity. Amoxicillin and erythromycin had no obvious inhibitory effects on the two bacteria, indicating that the bacteria were resistant against the antibiotics. The camelliagenin plus antibiotics had better inhibition on bacteria than the camelliagenin or antibiotics alone, showing that the camelliagenin can strengthen bacterial sensitivity to antibiotics (Table 1).

**Table 1:** Inhibition concentration (μg/ml) of the saponin extract on 50 % bacterial growth and biofilm formation of *Escherichia coli* and *Staphylococcus aureus*

Groups	*E. coli*		*S. aureus*	
	Growth	Biofilm	Growth	Biofilm
Amo	72.6 ± 7.9	72.8 ± 9.6	138.6 ± 11.0	129.8 ± 10.5
Sap	50.2 ± 5.7^a^	43.6 ± 4.2^a^	79.6 ± 9.1^a^	70.2 ± 10.2^a^
Sap : Amo (1:1)	30.4 ± 6.5^a,b^	22.8 ± 5.2^a,b^	59.0 ± 9.6^a,b^	52.4 ± 8.0^a,b^
Sap : Amo (5:1)	23.4 ± 5.6^a,b^	18.6 ± 5.7^a,b^	43.2 ± 5.9^a,b^	39.2 ± 3.5^a,b^
Sap : Amo (10:1)	21.6 ± 5.1^a,b^	15.6 ± 3.5^a,b^	35.2 ± 4.5^a,b^	30.2 ± 7.0^a,b^
Ery	69.2 ± 6.3	65.6 ± 10.5	126.2 ± 6.7	122.8 ± 8.3
Sap : Ery (1:1)	28.2 ± 5.2^b,c^	26.8 ± 6.8^b,c^	51.6 ± 8.0^b,c^	44.4 ± 6.1^b,c^
Sap : Ery (5:1)	24.4 ± 4.7^b,c^	19.2 ± 4.8^b,c^	35.4 ± 5.3^b,c^	28.6 ± 6.3^b,c^
Sap : Ery (10:1)	13.4 ± 3.9^b,c^	12.8 ± 5.1^b,c^	27.6 ± 5.7^b,c^	22.8 ± 6.2^b,c^

Data were presented as mean ± standard deviation (*n* = 5)

*Amo* amoxicillin, *Sap* saponin, *Ery* erythromycin

^a^ *p* < 0.01, compared with amoxicillin

^b^ *p* < 0.01, compared with the saponin

^c^ *p* < 0.01, compared with erythromycin

**Fig. 4:**
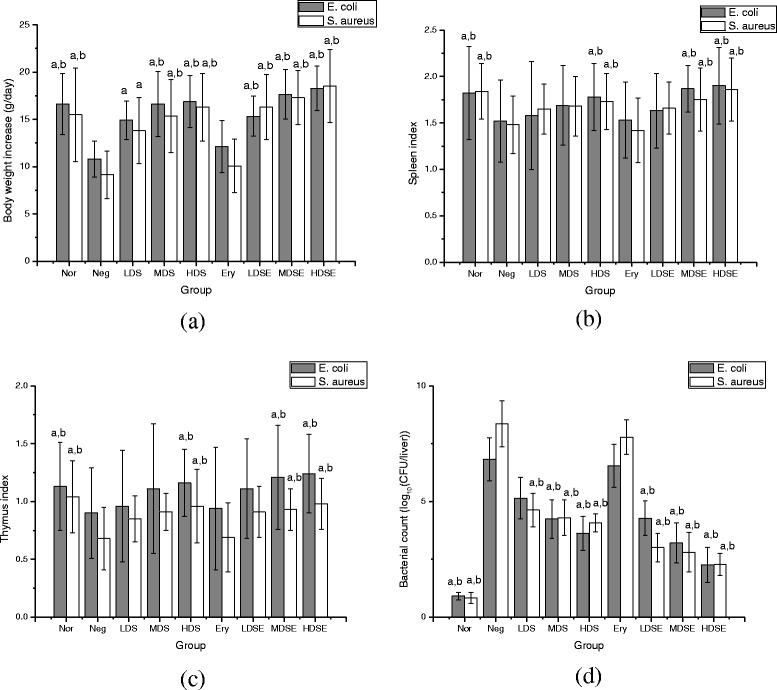
The effects of the saponin on body weight increase (**a**), spleen index (**b**), thymus index (**c**) and bacterial count (**d**) in liver of chicks infected by *Escherichia coli* and *Staphylococcus aureus.* Chicks in each group were injected intraperitoneally by 0.2 ml (1 × 10^8^ CFU/ml) of *E. coli* or *S. aureus* suspension except normal group, 10 h later administered with feed containing erythromycin (50 mg/kg) or the saponin (50 mg/kg in low dose, 250 mg/kg in middle dose, 500 mg/kg in high dose) respectively for consecutive 3 d. The chicks were weighed, monitored for 14 d. a, *p* < 0.01, compared with negative group; b, *p* < 0.01, compared with erythromycin group. Nor: normal group; Neg: negative group; LDS: low dose of the saponin; MDS: middle dose of the saponin; HDS: high dose of the saponin; Ery: erythromycin; LDSE: low dose of the saponin + erythromycin; MDSE: middle dose of the saponin + erythromycin; HDSE: high dose of the saponin + erythromycin

Inhibition of the camelliagenin on bacteria *in vivo* was evaluated by its therapeutic effect on chicks induced by *E. coli* and *S. aureus*. Chicks in negative groups showed serious symptoms of depression, anorexia, dullness, and diarrhea. Amoxicillin and erythromycin did not relieve these symptoms obviously, suggesting bacterial resistance against the antibiotics. The camelliagenin and combined administration of the camelliagenin and antibiotics significantly (*p* < 0.01) increased the body weight, immune organ index and reduced bacterial counts of liver in dose dependence, indicating that the camelliagenin enhances bacterial sensitivity to antibiotics and improves chicks immunity. The results are shown in Fig. 4. It further proves that the camelliagenin can not only substitute antibiotics, but also enhance antibiotic effects.

In order to reduce the number of experimental animals and limitation of models, inhibitory tests were both carried out on bacteria *in vitro* and *in vivo*. The results show positive effects on inhibition of bacterial growth, indicating that the camelliagenin is promising as an effective antibacterial agent for *E. coli* and *S. aureus*. It may take effects on other kinds of bacteria, but needs further evaluation.

### Effects of the camelliagenin on biofilm formation and exudates

Bacterial biofilm is a key factor to induce resistance against antibiotics and a target of new antimicrobials [17]. Thus the biofilms of *E. coli* and *S. aureus* were analyzed in our research. The results showed that amoxicillin or erythromycin had no different effect on biofilms from negative group (Table 1), and IC_50_ of the camelliagenin on biofilm of the two bacteria were lower than that of amoxicillin or erythromycin at significant level (*p* < 0.01). It indicates that antibacterial effects of the camelliagenin are related to its inhibition on bacterial biofilm formation.

The expression of specific genes is involved in biofilm formation and responsible for bacterial drug resistance [18]. The main difference between biofilm bacteria and planktonic bacteria is that biofilm bacteria are tightly packed and wrapped in their own secreted extracellular polysaccharide matrix called extracellular polymeric substances (EPS). The main component of EPS is alginate [19]. Mannitol dehydrogenase is a key enzyme in alginate synthesis process of biofilm [20]. Some antimicrobials can destroy biofilm formation since it inhibits activity of mannitol dehydrogenase in biosynthetic pathway of alginate [21]. In addition, large amount of extracellular DNA (eDNA) is found in the biofilm [22], it not only affects the formation of biofilms, but also increases the resistance of biofilms by chelating cation [23]. It is proved that eDNA enzymes can clear immature biofilm *in vitro* [24], and biofilm formation can be regulated by mannitol dehydrogenase and eDNA [25].

**Fig. 5:**
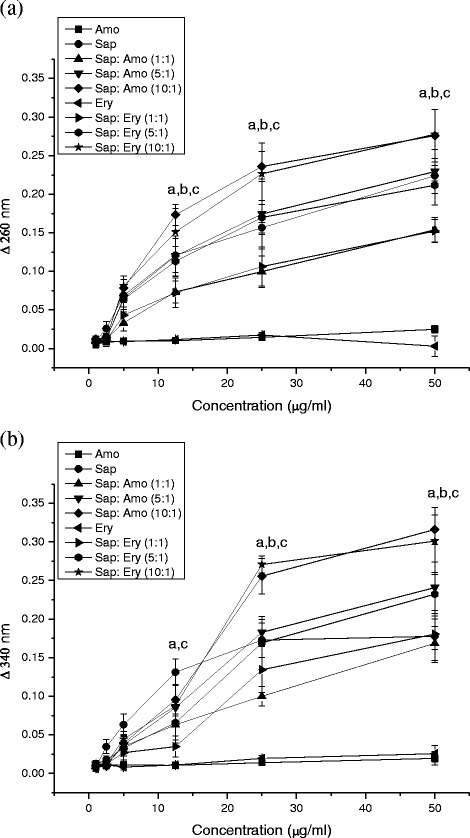
Activity of mannitol dehydrogenase (**a**) and content of extracellular DNA (b) in bacterial biofilm affected by the saponin and antibiotics ($\overline{x}+s$, *n* = 5). Data were measured respectively by a decrease in the absorbance of reactive mixture at 340 nm and biofilm lysate at 260 nm compared to untreated controls. ∆ 340 nm reflects decrease of mannitol dehydrogenase activity, and ∆ 260 nm reflects decrease of eDNA in biofilm. a, *p* < 0.01, compared with amoxicillin; b, *p* < 0.01, compared with the saponin; c, *p* < 0.01, compared with erythromycin. Amo: amoxicillin; Sap: saponin; Ery: erythromycin

MDH and eDNA in biofilm were measured respectively by a decrease in the absorbance of reactive mixture at 340 nm and biofilm lysate at 260 nm compared to untreated controls. ∆A_340_ and ∆A_260_ increased significantly (*p* < 0.01) after the treatments of the camelliagenin at concentration dependence (Fig. 5). It suggests that they play a role in the inhibition of MDH and eDNA in the biofilms.

### Interaction of the camelliagenin with mannitol dehydrogenase and eDNA

**Fig. 6:**
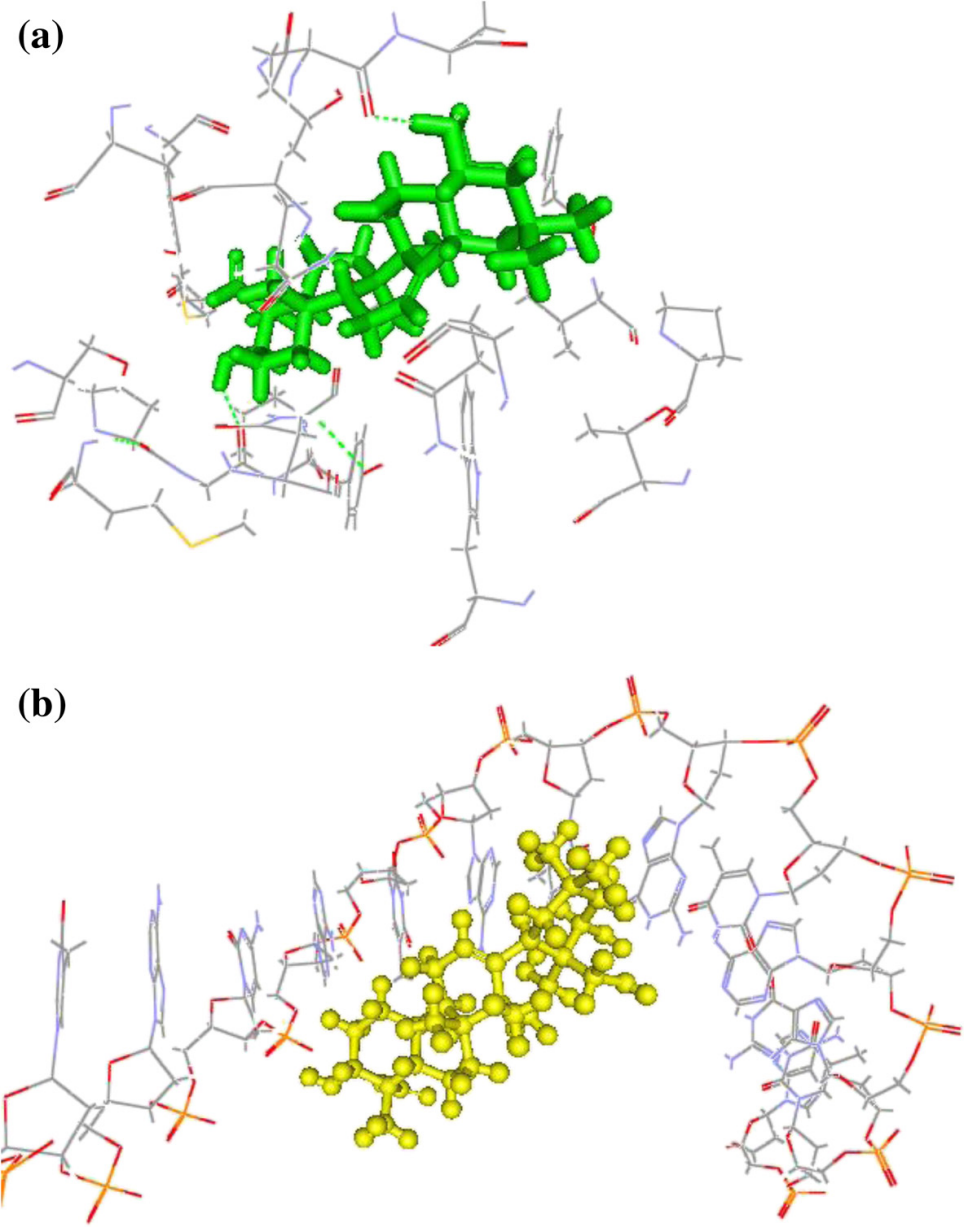
Interaction of the saponin with mannitol dehydrogenase (**a**) and extracellular DNA (**b**)

Molecular docking as a better method of molecular simulation is applied in this research. Docking simulation shows that the saponin can interact with mannitol dehydrogenase (MDH) and eDNA. Each molecule successfully docks in 10 poses. The average binding energy is separately −86.94 ± 1.99 kcal/mol and −105.01 ± 1.19 kcal/mol, the average interactive energy is respectively 39.70 ± 2.28 kcal/mol and 19.77 ± 1.64 kcal/mol. It suggests that the camelliagenin can spontaneously bind to MDH and eDNA and exerts stronger interaction. Mimic diagrams of the camelliagenin binding to mannitol dehydrogenase and DNA are shown in Fig. 6, illustrating that they can well bind and interact with each other.

Based on the lock-key principle and complementary structural hypothesis, molecular docking simulates mutual interaction between ligand and receptor [26]. If receptor and ligand can interact, they must approach each other, and then combine in a particular conformation of the binding site, finally reach stable complex by adjusting conformation. Correct affinity prediction is conducive to drug design and screening. The main factors affecting the binding stability of ligand and receptor are hydrophobic force and bonding force. Free energy value is an important parameter for evaluation of docking affinity, binding activity and stability of receptor and ligand, and it can be used to judge the interaction of ligand and receptor. We simulated the interaction of the camelliagenin with mannitol dehydrogenase and eDNA, the result shows a good correlation between the interaction and its anti-biofilm effect. It suggests that molecular docking can be used to predict the saponin effect on biofilm inhibition.

Saponins are detergent-like substances showing antibacterial potential, and the mechanism deserves discussion. The critical micelle concentration of the camelliagenin is 0.5 % [27], but its MIC_90_ and IC_50_ are far away from the concentration whereas it shows detergent activity, demonstrating that its antibacterial activity is not due to the surface property. It is reported that the effect of saponin is associated with the changing of membrane permeability of Gram-negative cells in contrast to Gram-positive cells. Saponin might interact with Gram-negative cells components, like lipid A and thereby increase the permeability of bacterial cell wall [28]. Because the camelliagenin can reduce mannitol dehydrogenase and eDNA in biofilm, we provide a new hypothesis that the camelliagenin interacts with key components in biofilm, and blocks their activities in biofilm formation. The interaction of the camelliagenin with mannitol dehydrogenase and eDNA contributes to its anti-biofilm activity.

## Conclusions

The camelliagenin is isolated from the defatted seeds of *C. oleifera* by ultrasonic assisted acid–base alternative technique. This is cost effective for industrialization because no organic solvent and expensive equipment are applied in the process. It is identified as camelliagenin and has significant antibacterial activities on *E. coli* and *S. aureus in vitro* and *in vivo*. It suggests that the camelliagenin is hopeful candidate for prevention of antibiotic resistance. The effects of the camelliagenin include its inhibition on bacterial biofilm formation, which is related to MDH and eDNA in the biofilm. The camelliagenin can spontaneously bind to and interact with MDH and eDNA. That could be the mechanism of the camelliagenin action on the two bacteria.

## Methods

### Drugs and bacteria

The defatted seeds of *Camellia oleifera* Abel were collected from oil manufacturing company (Meizhou, China). *Escherichia coli* was collected from fecal samples of chicks with generalized colibacillosis in Guangdong Chicken Farm, and identified as amoxicillin-resistant *E. coli* using the reported method [29]. *Staphylococcus aureus* was collected from nasal swab species of chicks with staphylocosis in Guangxi Chicken Farm, and identified as erythromycin-resistant *S. aureus* by the method in the reference [30]. The fecal and nasal samples from the chickens were taken after we obtained consent from the farm owners. *E. coli* control strain ATCC25922 and *S. aureus* control strain ATCC25923 were purchased from Guangdong Microbial Institute (Guangzhou, China). Reagents for biofilm assay were NH broth, crystal violet, amoxicillin and erythromycin purchased from Shanghai Hualan Biochemical Company (Shanghai, China). Chemical reference substance camelliagenin was bought from Shanghai Chemicals Company (Shanghai, China). Other reagents were purchased from Guangzhou Reagent Company (Guangzhou, China).

### Animals

The experiments were carried out on ten-day-old male Roman chicks. The chicks were housed under conditions of 24 ± 2 °C, 50 ± 10 % humidity with a 12 h light/ dark cycle for 1 week adaptation. Food and water were accessible *ad libitum*. The experiments have been performed in accordance with the Chinese guidelines for the use of laboratory animals, and received approval from the animal experimentation ethic committee of South China University of Technology. All efforts were made to minimize animal suffering and to reduce the number of animals used. All sections of this report adheres to the ARRIVE guidelines for reporting animal research. A completed ARRIVE guidelines checklist is included in Checklist.

### Procedure of the isolation

1 kg of the defatted seeds of *C. oleifera* were crushed to pass through 20 mesh sieve, submerged in 2 % HCl aqueous solution (water / seeds = 20 / 1 in ml / g) with ultrasonic at 300 W for 1 h. In order to protect the compounds from heat, temperature was controlled below 60 °C with cooling water. The extract solution was stood for 5 h. The precipitate was washed with 1000 ml of 2 % NaOH and 500 ml of water, and dried in vacuum to get the saponin extract.

### Determination of purity by HPLC

The analysis of saponin was run on HP 1100 HPLC (Agilent Company, USA) in the following operating conditions: column: Hypersil ODS (250 × 4.6 mm, 5 μm); flow phase: methanol/water (80/20); injection volume: 10 μl; flow rate: 1 ml/min; temperature: 25 °C; wavelength: 209 nm [31].

### Structure determination

1 g of saponin extract was dissolved in 5 ml of 80 % methanol, and purified by silica gel chromatograph with elution by chloroform/methanol (8/2). The peaks were collected and dried in vacuum, and 0.5 g saponin was obtained for structural analysis.

UV spectra analysis was carried out on UV-3010 Ultra violet spectrometer (Hitachi Company, Japan) scanning from 200 to 600 nm. IR spectra were measured on Nicolet 380 FI-IR spectrograph (Nicolet Apparatus Company, USA) with KBr tablets from 4000 to 400 cm^−1^ with resolution 2 cm^−1^. Mass spectra were recorded on Bruker Esquire Hct Plus Mass spectrometer with ESI (Bruker Company, Germany) in m/z of cation model scanning from 150 to 1200 for 60 min. NMR spectra were determined on 400 MHz AM NMR (Bruker Company, Switzerland) in DMSO-d_6_ operating at 101 MHz for ^13^C NMR and 400 MHz for ^1^H NMR.

### Antibacterial activity

The antibacterial activity of the saponin against *E. coli* and *S. aureus* was examined using the microbroth dilution method according to the CLSI standard [32]. Thirty-five strains of *E. coli* and 30 strains of *S. aureus* were isolated using the method reported [33, 34], they were respectively inoculated in broth medium, which was cultured for 24 h at 37 °C, and diluted to 10^5^ CFU/ml. Serial dilutions were prepared from 500 μg/ml of the saponin using DMSO to make 500, 250, 125, 62.5, 31.25, and 15.625 μg/ml. The wells were inoculated with 0.1 ml aliquot of test bacteria (10^5^ CFU/ml) having serial dilutions of the saponin (50 μl, each). The micro plate was incubated at 37 °C ± 1 °C for 24 h. Dilution of the saponin to respective test organism showing no visible growth was considered as MIC. The minimum inhibitory concentration on 90 % bacterial strains was calculated as MIC_90_.

### Bacterial biofilm experiment

Bacterial biofilm experiments were carried out according to the reference [35]. The diluted bacteria (10^5^ CFU/ml) of *E. coli* and *S. aureus* were divided into the saponin groups, negative control (no drug), antibiotic groups (amoxicillin and erythromycin) and groups of the saponin plus antibiotics (1:1, 5:1, 10:1) with 5 repetition wells for each. The extracts were dissolved with water to 500 μg/ml. 50 μl of drug solution and 450 μl of diluted bacteria (final drug concentration 50 μg/ml) were mixed in one well of 24 well plates, cultured for 24 h, and then absorbance in 490 nm (OD_490_) was measured with water as blank to calculate the bacterial growth inhibition rate. The medium in the cell was carefully removed and washed 3 times with 1 ml of distilled water, and 1 % crystal violet solution (500 μl) was added to dye the biofilm. 30 min later, the solution was aspirated, and 500 μl of ethanol was added to extract crystal violet from the biofilm. The absorbance in 560 nm (OD_560_) was determined with ethanol as blank to calculate the biofilm inhibition rate.

$$ \mathrm{Bacterial}\ \mathrm{growth}\ \mathrm{inhibtion}\ \mathrm{rate}\ \left(\%\right) = \frac{\left(\mathrm{Negative}\ {\mathrm{OD}}_{490}-\mathrm{Drug}\ {\mathrm{OD}}_{490}\right)}{{\mathrm{Negative}\ \mathrm{O}\mathrm{D}}_{490}}\times 100\% $$

$$ \mathrm{Biofilm}\ \mathrm{inhibition}\ \mathrm{rate}\ \left(\%\right) = \frac{\left(\mathrm{Negative}\ {\mathrm{OD}}_{560}-\mathrm{Drug}\ {\mathrm{OD}}_{560}\right)}{{\mathrm{Negative}\ \mathrm{O}\mathrm{D}}_{560}}\times 100\% $$

Inhibitory concentrations (IC_50_) of the drugs on 50 % bacterial growth and biofilm formation were determined by dilution until the inhibition rate was 50 %. It is used as the index to evaluate the inhibitory effects on bacteria.

### Assay of mannitol dehydrogenase and extracellular DNA in biofilm

The saponin extract and the antibiotics were diluted to final drug concentration in culture medium at 50, 25, 12.5, 5, 2.5, 1 μg/ml for 48 h bacteria culture. After removing the medium, the biofilm was homogenized in 500 μl of 50 mmol/l phosphate buffer (pH 5.5), 10 μl of the supernatant was taken for enzyme activity assay, and the rest was used for extracellular DNA determination.

Mannitol dehydrogenase (MDH) activity was determined according to a modified method [36]. Briefly, MDH was measured by the decrement of NADH, which was monitored by the absorbance at 340 nm. The reaction mixture contained 50 μl of 200 mM sodium phosphate buffer (pH 5.5), 50 μl of 2 mM NADH, 50 μl of water and 10 μl of the biofilm extract. The mixture was maintained at 32 °C for 2 min, and the reaction was started by adding 40 μl of 1 M fructose and lasted for 5 min. The absorbance at 340 nm was detected by UV-3010 spectrometer (Hitachi Company, Japan).

Extracellular DNA (eDNA) was measured in the following protocol [37]. The biofilm extract was mixed with 10 U/ml cellulase at 37 °C for 1 h, followed by treatment with 10 U/ml proteinase K for another 1 h. Treated samples were centrifuged at 10, 000 g for 10 min. The supernatant was collected and the absorbance was detected at 260 nm by UV spectrometer to deduce eDNA content in biofilm.

### Antibacterial test *in vivo*

The antibacterial tests were performed as the reported with some modification [38]. Two tests including *E. coli* and *S. aureus* were carried out separately. For one test, the chicks were randomly divided into 9 groups with 30 chicks in each group, including normal group, negative group, antibiotic group, the saponin extract and the saponin extract plus antibiotic groups in high, middle and low dose. The groups of chicks except normal group were injected intraperitoneally with 0.2 ml of 1 × 10^8^ CFU/ml bacterial suspension, 10 h later they were administered with 1 kg of feed containing 50 mg amoxicillin or erythromycin in antibiotic group, 50 mg, 250 mg and 500 mg in low, middle and high dose of the saponin extract respectively for consecutive 3 d. The doses were based on regular use of antibiotics and safety of the saponin [39]. The chicks were weighed, monitored for 14 d, and evaluated by relief of symptoms of anorexia, lassitude, diarrhea, etc. [40]. The chicks were euthanized on the fifteenth day, thymus and spleen were removed and weighed to calculate immune organ index (organ weight / body weight). Chick liver was plated onto Tryptose Soya Agar (TSA) plates for bacterial colony count, and the bacterial density was expressed as the number of CFU per liver.

### Simulation of molecular interaction

Molecular docking was simulated by Discovery Studio V2.5 software (Accelry Inc., CA, USA) in the following procedure [41]. Structural data of mannitol dehydrogenase and eDNA were downloaded from Brookhaven Library. The missing amino acids and hydrogens were supplemented, and excessive protein conformation was removed. Mannitol dehydrogenase and eDNA were separately defined as the receptor, and then binding sites and coordinates were defined. A new plot window was open to draw structure of the extracts, optimize 3 D geometric structures, and apply CHARMm force field to ensure correct bond length, bond angle in a state of energy stability. CDOCKER Protocol was run to obtain binding parameters, which are used to evaluate the interaction. CDOCKER energy and CDOCKER interaction energy are two important parameters, and their absolute values are in according with the affinity and action force between the receptor and ligand.

### Statistical analysis

Data was presented as mean ± standard deviation ($\overline{x}\pm s$). SPSS 11.0 software (SPSS Inc., USA) was used to analyze the data of animal tests in groups by one-way ANOVA and Dunnett’s test.
